# AcABI5a integrates abscisic acid signaling to developmentally modulate fruit ascorbic acid biosynthesis in kiwifruit

**DOI:** 10.1093/hr/uhaf111

**Published:** 2025-04-24

**Authors:** Xiaoying Liu, Yachen Li, Xianzhi Zhang, Xiaodong Xie, Abu Naim Md Muzahid, Jing Tu, Lansha Luo, Gudeta Chalchisa, Haiyan Lv, Hua Tian, Sean M Bulley, Dawei Li, Caihong Zhong

**Affiliations:** CAS Key Laboratory of Plant Germplasm Enhancement and Specialty Agriculture, Wuhan Botanical Garden, The Innovative Academy of Seed Design, Chinese Academy of Sciences, No. 201, Jiufeng 1st Road, Donghu Hi-Tech Development Zone, Wuhan 430074, Hubei, China; State Key Laboratory of Plant Diversity and Specialty Crops, Wuhan Botanical Garden, Chinese Academy of Sciences, No.201, Jiufeng 1st Road, Donghu High-Tech Development Zone, Wuhan, Hubei, 430074, China; Zhongkai University of Agriculture and Engineering, 24 Dongsha Street, Haizhu District, Guangzhou City, Guangdong Province, 510225, China; Zhongkai University of Agriculture and Engineering, 24 Dongsha Street, Haizhu District, Guangzhou City, Guangdong Province, 510225, China; CAS Key Laboratory of Plant Germplasm Enhancement and Specialty Agriculture, Wuhan Botanical Garden, The Innovative Academy of Seed Design, Chinese Academy of Sciences, No. 201, Jiufeng 1st Road, Donghu Hi-Tech Development Zone, Wuhan 430074, Hubei, China; State Key Laboratory of Plant Diversity and Specialty Crops, Wuhan Botanical Garden, Chinese Academy of Sciences, No.201, Jiufeng 1st Road, Donghu High-Tech Development Zone, Wuhan, Hubei, 430074, China; CAS Key Laboratory of Plant Germplasm Enhancement and Specialty Agriculture, Wuhan Botanical Garden, The Innovative Academy of Seed Design, Chinese Academy of Sciences, No. 201, Jiufeng 1st Road, Donghu Hi-Tech Development Zone, Wuhan 430074, Hubei, China; State Key Laboratory of Plant Diversity and Specialty Crops, Wuhan Botanical Garden, Chinese Academy of Sciences, No.201, Jiufeng 1st Road, Donghu High-Tech Development Zone, Wuhan, Hubei, 430074, China; College of Life Science, Nanchang University, 999 Xuefu Avenue, Honggutan District, Nanchang, Jiangxi, 330031, China; Zhongkai University of Agriculture and Engineering, 24 Dongsha Street, Haizhu District, Guangzhou City, Guangdong Province, 510225, China; CAS Key Laboratory of Plant Germplasm Enhancement and Specialty Agriculture, Wuhan Botanical Garden, The Innovative Academy of Seed Design, Chinese Academy of Sciences, No. 201, Jiufeng 1st Road, Donghu Hi-Tech Development Zone, Wuhan 430074, Hubei, China; State Key Laboratory of Plant Diversity and Specialty Crops, Wuhan Botanical Garden, Chinese Academy of Sciences, No.201, Jiufeng 1st Road, Donghu High-Tech Development Zone, Wuhan, Hubei, 430074, China; CAS Key Laboratory of Plant Germplasm Enhancement and Specialty Agriculture, Wuhan Botanical Garden, The Innovative Academy of Seed Design, Chinese Academy of Sciences, No. 201, Jiufeng 1st Road, Donghu Hi-Tech Development Zone, Wuhan 430074, Hubei, China; State Key Laboratory of Plant Diversity and Specialty Crops, Wuhan Botanical Garden, Chinese Academy of Sciences, No.201, Jiufeng 1st Road, Donghu High-Tech Development Zone, Wuhan, Hubei, 430074, China; CAS Key Laboratory of Plant Germplasm Enhancement and Specialty Agriculture, Wuhan Botanical Garden, The Innovative Academy of Seed Design, Chinese Academy of Sciences, No. 201, Jiufeng 1st Road, Donghu Hi-Tech Development Zone, Wuhan 430074, Hubei, China; State Key Laboratory of Plant Diversity and Specialty Crops, Wuhan Botanical Garden, Chinese Academy of Sciences, No.201, Jiufeng 1st Road, Donghu High-Tech Development Zone, Wuhan, Hubei, 430074, China; Kiwifruit and Subtropicals Physiology Team, The New Zealand Institute for Plant and Food Research Limited, 412 No 1 Rd, RD2, Te Puke 3182, New Zealand; CAS Key Laboratory of Plant Germplasm Enhancement and Specialty Agriculture, Wuhan Botanical Garden, The Innovative Academy of Seed Design, Chinese Academy of Sciences, No. 201, Jiufeng 1st Road, Donghu Hi-Tech Development Zone, Wuhan 430074, Hubei, China; State Key Laboratory of Plant Diversity and Specialty Crops, Wuhan Botanical Garden, Chinese Academy of Sciences, No.201, Jiufeng 1st Road, Donghu High-Tech Development Zone, Wuhan, Hubei, 430074, China; CAS Key Laboratory of Plant Germplasm Enhancement and Specialty Agriculture, Wuhan Botanical Garden, The Innovative Academy of Seed Design, Chinese Academy of Sciences, No. 201, Jiufeng 1st Road, Donghu Hi-Tech Development Zone, Wuhan 430074, Hubei, China; State Key Laboratory of Plant Diversity and Specialty Crops, Wuhan Botanical Garden, Chinese Academy of Sciences, No.201, Jiufeng 1st Road, Donghu High-Tech Development Zone, Wuhan, Hubei, 430074, China

## Abstract

Consumers value highly the nutritional content and flavor of fresh fruits, which are influenced by endogenous plant hormones. However, the molecular mechanisms governing the hormonal regulation of essential nutrients such as ascorbic acid (AsA) in fruit are still unclear. This study investigates the regulation of AsA synthesis in kiwifruit by the transcription factor AcABI5a, which is involved in mediating the abscisic acid (ABA) signal. A negative correlation between *AcABI5a* expression and AsA levels across different developmental stages of kiwifruit was observed. Furthermore, AcABI5a was found to bind both the *AcMYBS1* promoter, repressing its transcriptional activity, and its own promoter, fostering expression and maintaining active repression of *AcMYBS1*. AcMYBS1 activates the expression of *AcGGP3*, which encodes an enzymatic step in AsA biosynthesis that is highly regulated both transcriptionally and translationally. In-depth interaction studies utilizing yeast two-hybrid (Y2H), bimolecular fluorescence complementation (BiFC), firefly luciferase complementation (NC-LUC), and pull-down assays unveiled that AcABI5a also physically interacts with AcMYBS1, further impeding its activation of *AcGGP3*. Results from knockout by gene editing and overexpression of *AcABI5a* support the role of AcABI5a in mediating the ABA inhibitory effect on AsA synthesis by repressing the expression of *AcMYBS1* and thus *AcGGP3*. Overall, our findings highlight AcABI5a’s negative regulatory role in AsA synthesis by integrating ABA signaling during fruit development, providing new insights into the regulation of AsA synthesis by phytohormones.

## Introduction

L-ascorbic acid (AsA), commonly known as vitamin C, is a vital nutritional component and antioxidant that functions as a critical redox buffer and enzyme cosubstrate in eukaryotes. In plants, AsA is pivotal for scavenging reactive oxygen species (ROS) generated by biotic and abiotic stresses, as well as by photosynthesis and respiration, thus facilitating the maintenance of normal growth-related physiological processes [1]. Moreover, AsA is essential for human well-being and disease prevention. Given that humans and certain other primates lack the ability to synthesize their own AsA, they rely on dietary sources such as fresh fruits and vegetables for its acquisition [2]. Numerous studies have robustly demonstrated the health benefits of AsA-rich diets derived from fruits and vegetables, continually highlighting the significance of AsA metabolism in horticultural crops as a primary target for nutritional enhancement [3].

Kiwifruit (includes various *Actinidia* species) are renowned for their abundant levels of AsA, polyphenols, and other health-promoting metabolites [4]. The L-galactose pathway serves as the primary route for AsA accumulation in higher plants, with GDP-L-galactose phosphorylase (GGP) identified as the pivotal rate-limiting enzyme for AsA synthesis in kiwifruits [5]. Notably, the overexpression of kiwifruit *GGP* in tomato, strawberry, and potato has resulted in a remarkable 2- to 6-fold increase in AsA levels in the fruits of tomatoes and strawberries, as well as in potato tubers [5]. Furthermore, both transient and stable transformations of *Arabidopsis* and *Nicotiana benthamiana* with the kiwifruit *GGP* gene have demonstrated the synergistic regulation of AsA biosynthesis by *GGP*, in conjunction with GDP-D-mannose-3′, 5′-epimerase (*GME*) [5]. Comparative studies within the *Actinidia* genus, particularly between high-AsA species (*Actinidia eriantha* and *Actinidia latifolia*) and low-AsA species (*Actinidia rufa*), have revealed a higher expression of the *GGP3* gene in species with elevated AsA levels, exhibiting a positive correlation with AsA content [4, 6]. However, it is essential to recognize that AsA synthesis is influenced not only by genetic regulation and redox status, but also by phytohormones [6–9] and environmental factors such as temperature [10] and light [11–14]. Therefore, investigating how AsA is regulated by these internal plant factors and external environmental conditions emerges as a critical aspect in understanding the AsA metabolism of kiwifruit.

Abscisic acid (ABA) is an important phytohormone that plays a vital role in numerous aspects of plant growth, development, carbohydrate anabolism, and stress responses [15–17]. ABA can induce ROS accumulation and participate in plant stress response signaling [16, 18]. The ABA signaling pathway comprises the ABA receptor-pyrabactin resistance/pyrabactin resistance-like/regulatory components of the ABA receptor (PYR/PYL/RCAR), type 2C protein phosphatase (PP2C), SNF1-related protein kinase 2 (SnRK2), and transcription factors (TFs) ABF/AREB [19–21]. The *ABSCISIC ACID INSENSITIVE* (*ABI*) genes encode AP2/REF TFs, which are transcriptionally activated and recognized as key elements in the ABA signaling pathway [15, 22–24]. ABIs are involved in various plant development processes, including seed germination, response to abiotic stress [25–27], auxin-mediated signaling, lateral root development [28], AsA and sugar biosynthesis [28, 29], and the chloroplast-to-nuclear retrograde signaling pathway [30, 31]. This broad spectrum of functions highlights the extensive regulatory roles played by ABI TFs in plants.

AsA biosynthesis in plants is regulated by phytohormones, such as ABA, auxin, salicylic acid (SA), jasmonic acid (JA), and ethylene. Specifically, auxin and ABA exert antagonistic effects on AsA synthesis and drought tolerance during tomato development [7, 32]. In *Arabidopsis*, the interplay between ABA signaling and AsA synthesis pathways, mediated by *PTP-like Nucleotidase* (*PTPN*), has been shown to enhance drought tolerance [33]. Low AsA content triggers signaling via ABA, SA, and JA dependent in an ABI4-dependent manner, collectively orchestrating plant growth and defense mechanisms [34]. Conversely, exogenous AsA treatment elicits ABA, auxin, and JA production [35].

Exogenous ABA treatment of *Arabidopsis* leaves significantly reduced AsA accumulation while supplementation with 1-aminocyclopropane-1-carboxylic acid (ACC), an ethylene precursor, restored AsA concentrations, indicating that ethylene antagonized the inhibitory effect of ABA on plant AsA biosynthesis [9]. Indeed, AsA is a cosubstrate of ACC oxidase, the rate-limiting step in ethylene biosynthesis [36]. Moreover, in *Arabidopsis*, ABI4 inhibited the expression of *GGP*, thereby reducing AsA synthesis [8, 9]. Our recent studies have shown that ABA limits AsA synthesis by downregulating 1R-subtype myeloblastosis protein (*AcMYBS1*) expression. In addition, the AsA content in several kiwifruit species decreased progressively with the growth period [6]. However, whether this was regulated by ABA remained to be more fully explored.

In this study, we elucidate the mechanism by which *AcABI5a*, acting through ABA signaling, suppresses the expression of *AcMYBS1*. Our findings indicate that following ABA exposure in kiwifruit, *AcABI5a* expression is stimulated, and then AcABI5a self-activates its own expression as well as binding to the promoter of *AcMYBS1* to repress expression of *AcMYBS1*. Furthermore, AcABI5a interacts with AcMYBS1 to impede AcMYBS1 binding to the promoter of *AcGGP3*. By employing *AcABI5a* overexpression and gene editing, we demonstrate the effects on AsA biosynthesis mediated by AcABI5a, thereby elucidating the molecular mechanism by which ABA regulates the AsA concentration in fruit over development.

## Results

### ABA inhibits AsA synthesis in kiwifruit

**Figure 1:**
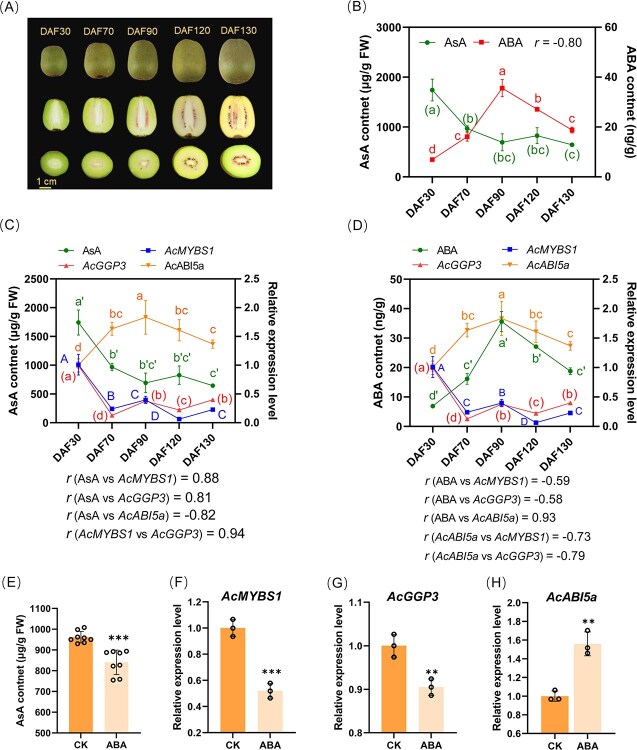
The biosynthesis of ascorbic acid (AsA) in kiwifruit is modulated by the ABA concentration across fruit developmental stages, with ABA inducing *AcABI5a* expression and inhibiting *AcMYBS1* expression. (A) Fruit morphology and cross sections of ‘Donghong’ over different developmental stages. (B) Concentration and correlation (*r*) between AsA and ABA in ‘Donghong’ fruits over different developmental stages. (C) RT-qPCR and correlations of the gene expression between *AcMYBS1*, *AcGGP3, AcABI5a*, and AsA content in (B). (D) Correlation between ABA content and *AcMYBS1*, *AcGGP3*, and *AcABI5a* expression in (B). (E) AsA content of ‘Donghong’ fruits that were injected with 10 μM ABA (ABA) or pure water (CK), and the experiment was repeated three times. (F)–(H) Gene expression level (by RT-qPCR) of (F) *AcMYBS1*, (G) *AcGGP3*, and (H) *AcABI5a* in (E). ^*^ Indicates that *AcABI5b* was barely detected. Values are means ±SD of at least three independent biological replicates. Significant differences were detected by *t*-test (^**^P < 0.01; ^***^P < 0.001). Different letters above the bars indicate significant differences among different groups at the *P* < 0.05 level obtained by the one-way ANOVA test. All correlations referred to are Pearson correlation coefficients.

The AsA content in *Actinidia chinensis var. chinensis* ‘Donghong’ fruits exhibited variations across different developmental stages, with the highest levels observed during early stages, reaching a nadir at DAF90, followed by stabilization (Fig. 1A and B). As was found previously in different genotypes [6], the expression profiles of *AcMYBS1* and *AcGGP3* displayed strong positive correlations with AsA content (*r* = 0.88 and *r* = 0.81, respectively) (Fig. 1C). In contrast, ABA levels exhibited an inverse trend, with the lowest concentrations detected during early developmental stages, peaking at DAF90, and subsequently declining gradually (Fig. 1B). This pattern demonstrates an inverse synthesis trend and a negative correlation with both AsA content (*r* = −0.8) and the expression of *AcMYBS1* (*r* = −0.59) and *AcGGP3* (*r* = −0.58) (Fig. 1B and D). Under typical growth conditions in kiwifruit, this observation suggests that ABA may have a negative regulatory role on AsA biosynthesis in fruit tissues. To test this hypothesis, 10 μM ABA (concentration derived from earlier work [6]) was injected into isolated kiwifruit, which were then placed at room temperature and normal humidity. After 3 days, AsA content in ABA-injected fruits decreased by 12.5% compared with the untreated control (Fig. 1E). Moreover, the expression levels of *AcMYBS1* and *AcGGP3* were also significantly reduced (Fig. 1F and G) while *AcABI5a* expression was upregulated by ABA treatment (Fig. 1H), suggesting that ABA suppresses AsA biosynthesis in the fruit of kiwifruit.

### ABA upregulates *AcABI5a* but downregulates *AcMYBS1* expression

To explore candidate genes involved in the ABA-mediated regulation of AsA biosynthesis in kiwifruit, we performed a transcriptomic analysis on ‘Donghong’ before and after ABA treatment, identifying 862 downregulated and 445 upregulated differentially expressed genes (DEGs) (Supplementary Fig. S1A). Gene Ontology (GO) enrichment analysis of these DEGs highlighted a preponderance in the category of ‘nucleic acid-binding TF activity’ (Supplementary Fig. S1B). Subsequent examination of the expression profiles of genes associated with AsA synthesis and ABA signaling pathways (Supplementary Table S1) revealed a notable decrease in *AcGGP3* and *AcMYBS1* levels post-ABA treatment, while the expression of an *ABI5-like* gene (named *AcABI5a* herein), displayed a significant increase, surpassing that of other ABI-like genes named *AcABI5b* and *AcABI3a* (Supplementary Fig. S1C). Upon further examination of the gene expression profiles of AsA synthesis and ABA signaling pathways (Supplementary Table S1) in ‘Donghong’ fruit over development [37], it was discovered that the expression of *AcGGP3* and *AcMYBS1* reached its peak during the initial stages of fruit development and decreased during the later stages (Supplementary Fig. S2). Notably, *AcABI5b* expression was nearly negligible across all stages, whereas *AcABI5a* and *AcABI3a* expression increased during the mid-period, with *AcABI5a* reaching its apex around DAF95, coinciding with the highest ABA content in ‘Donghong’ fruit at DAF90 (Fig. 1B and Supplementary Fig. S2). Similarly, real-time qualitative polymerase chain reaction (RT-qPCR) analysis indicated that the expression level of *AcABI5a* peaked at DAF90 and exhibited a high correlation coefficient of 0.93 with ABA content (Fig. 1D). Conversely, it showed significant negative correlations with *AcMYBS1* (*r* = −0.73), *AcGGP3* (*r* = −0.79), and AsA content (*r* = −0.82) across different developmental stages (Fig. 1C and D). Subcellular localization assays revealed that AcABI5a is localized in the nucleus (Supplementary Fig. S3). These findings suggest that AcABI5a plays an important role in the ABA-induced suppression of AsA biosynthesis in the fruit of kiwifruit.

### *AcABI5a* negatively regulates AsA synthesis in kiwifruit

**Figure 2:**
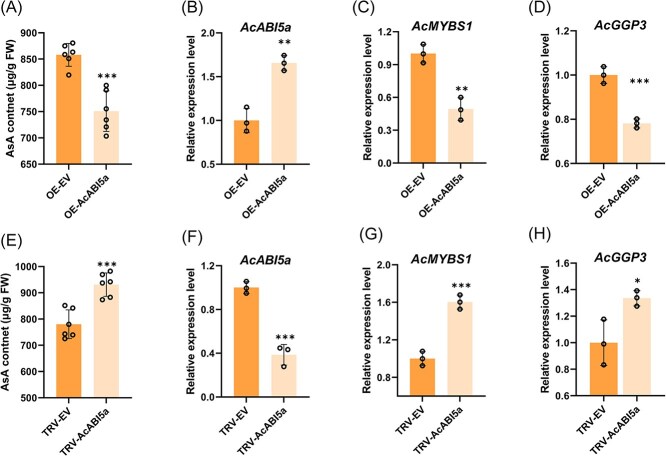
AcABI5a negatively regulates ascorbic acid (AsA) synthesis in transiently transformed kiwifruit (fruit tissue). (A) AsA content of ‘Donghong’ fruit after 5 days of transient *AcABI5a* overexpression (OE, *n* = 6), EV: empty vector. (B)–(D) Gene expression (by RT-qPCR) of (B) *AcABI5a*, (C) *AcMYBS1*, and (D) *AcGGP3* in (A). (E) AsA content of ‘Donghong’ fruit after 5 days of transient *AcABI5a* antisense-expression (TRV, *n* = 6). (F)–(H) Gene expression (by RT-qPCR) of (F) *AcABI5a*, (G) *AcMYBS1*, and (H) *AcGGP3* in (E). These experiments were repeated three times. Values are means ±SD of at least three independent biological replicates. Significant differences were detected by *t*-test (^*^P < 0.05; ^**^P < 0.01; ^***^P <0.001).

To elucidate the regulatory role of *AcABI5a* in AsA synthesis in kiwifruit, we initially performed transient expression assays in ‘Donghong’ fruits, which were infiltrated by *Agrobacterium tumefaciens* at DAF80. Five days postinfiltration, the fruit was sampled for measurement of AsA and gene expression (Fig. 2). AsA content in fruit overexpressing *AcABI5a* (OE-AcABI5a) was significantly reduced, while in fruit with *AcABI5a* silencing (TRV-AcABI5a), it was elevated compared with those infiltrated with the empty vector (EV) (Fig. 2A and E). Corresponding with the alterations in AsA content, in comparison to the EV treatment, the expression levels of *AcMYBS1* and *AcGGP3* were lower when *AcABI5a* was overexpressed and higher when *AcABI5a* was silenced (Fig. 2B–D and F–H).

**Figure 3:**
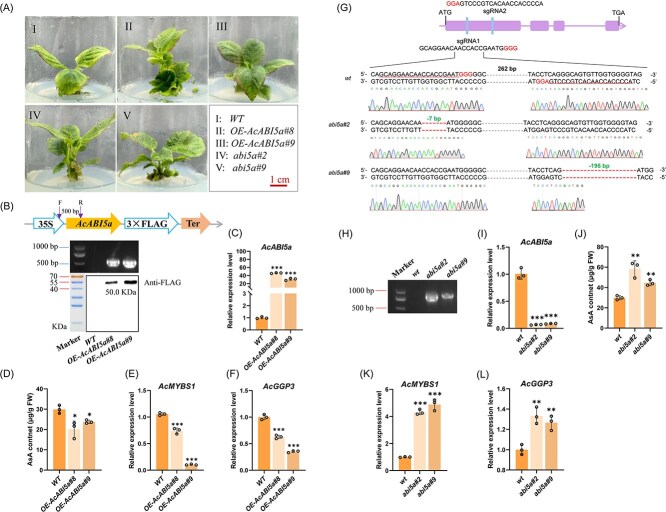
AcABI5a negatively regulates AsA synthesis in transgenic and gene editing kiwifruit lines. (A) Two *AcABI5a*-overexpression transgenic lines (*OE-AcABI5a#8* and *OE-AcABI5a#9*) and two independent *AcABI5a* homozygous mutant lines (*Abi5*#*2* and *Abi5*#*9*), *WT*: wild type. (B) The presence of the transgene in *AcABI5a*-overexpression lines was confirmed by PCR amplification and immunoblotting with FLAG antibody. The forward primer (F) for PCR amplification was derived from the 35S promoter sequence of the vector, and the reverse primer (R) was derived from the CDS sequence of *AcABI5a*. the length of the amplified fragment was 500 bp. The protein size of AcABI5a-3 × FLAG was 50.0 KDa. (C) RT-qPCR analysis gene expression level of *AcABI5a* of WT and wo *AcABI5a*-overexpression transgenic lines. (D) Leaf AsA content of *OE-AcABI5a#8* and *OE-AcABI5a#9* lines. (E)–(F) RT-qPCR analysis gene expression level of (E) *AcMYBS1* and (F) *AcGGP3* in (D). (G) A schematic map shown above represents the targeting sites in the exon regions (rectangle) of *AcABI5a*; the PAM motifs (NGG) are shown in the last three bases. Sequences and chromatograms of *AcABI5a* in ‘Donghong’ wild-type (*wt*) and two gene editing lines (*Abi5*#*2* and *Abi5*#*9*), reflecting the specifics of the gene editing events. The target sequence is underlined, the PAM sequences (GGG and GGA) can be seen, and the dashes indicate deletions. (H) Transgenic positive lines of editing of ‘Donghong’ were detected by agarose gel electrophoresis. The amplified fragment was a *Cas9-AcABI5a* vector fragment with a length of 930 bp. (I) Gene expression (by RT-qPCR) of *AcABI5a* in two gene editing lines. (J) Leaf AsA content of *AcABI5a* gene edited of ‘Donghong’ (note that fruit was not available for these lines). (K)–(L) Gene expression (by RT-qPCR) of (K) *AcMYBS1* and (L) *AcGGP3* in (J). Values are means ±SD of at least three independent biological replicates. Significant differences were detected by *t*-test (^*^P < 0.05; ^**^P < 0.01; ^***^P < 0.001).

Additionally, we confirmed the involvement of *AcABI5a* in AsA synthesis using ‘Donghong’ kiwifruit transgenic lines overexpressing *AcABI5a* or harboring gene-edited *AcABI5a* (Fig. 3A). The transgene in *AcABI5a-*overexpressing (*OE-AcABI5a#8* and *OE-AcABI5a#9*) leaves were detected by both PCR and immunoblotting (Fig. 3B). The *AcABI5a-*overexpressing lines exhibited significantly elevated *AcABI5a* expression (30- to 46-fold, Fig. 3C) and exhibited both reduced AsA content and expression of *AcMYBS1* and *AcGGP3* compared with wild-type (WT) (Fig. 3D–F). Correspondingly, two separate *AcABI5a*-edited lines (*Abi5#2* and *Abi5#9*) were detected and confirmed by sequencing (Fig. 3G and H). They were edited around sgRNA1 and sgRNA2, respectively (Fig. 3G and Supplementary Fig. S4). The two edited lines harbored 7-bp (*abi5a#2*) and 196-bp (*abi5a#9*) base pair deletions resulting in a substantial decrease in the expression level of *AcABI5a* (Fig. 3G and I). Both lines had significantly increased AsA content of tissue culture plantlet leaves compared with WT (Fig. 3J), along with marked upregulation of *AcMYBS1* and *AcGGP3* expression (Fig. 3K and L). The mutated allele in both lines resulted in frameshifts causing the formation of premature stop codons and thus truncated protein products. The 7-bp deleted *abi5a#2* resulted in 153 amino acid translated peptide and 196-bp deleted *abi5a#9* resulted in a 228 amino acid long peptide, compared with 418 amino acid WT peptide (Fig. 3F, Supplementary Fig. S5). Collectively, these results suggested a negative regulatory role of *AcABI5a* in AsA synthesis.

### AcABI5a serves as a dual regulator, inhibiting *AcMYBS1* transcription by binding to its promoter while simultaneously boosting its own transcription through binding to its own promoter

**Figure 4:**
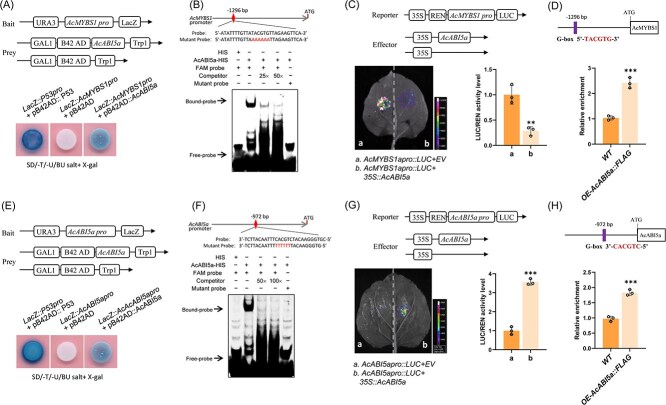
AcABI5a binds to promoters of both *AcMYBS1* and itself. (A) Y1H analysis, using *LacZ::AcMYBS1pro* as the bait, pB42AD::AcABI5a as the prey, *LacZ::P53pro,* with pB42AD::P53 serving as the positive control. (B) EMSA showing the interaction between fusion protein AcABI5a-HIS and the *AcMYBS1* promoter. The purified AcABI5a-HIS protein was incubated with the FAM-labeled probes containing G-box or mutated G-box elements. Unlabeled probe was used as a competitor. The bound DNA-protein complex is indicated by the arrows. +, presence; −, absence. (C) Dual-LUC assay using *AcMYBS1apro::LUC* as a reporter and *35S::AcABI5a* as the effector, *N. benthamiana* leaves were cotransformed with the reporter, and the empty effector vector as the negative control. The LUC/REN ratio of the control was normalized to 1, and a representative image was taken. (D) ChIP assay of *OE-AcABI5a::FLAG* transgenic kiwifruit leaves to show the binding of AcABI5a to the G-box (TACGTG) of *AcMYBS1* promoter. The chromatin was immunoprecipitated with FLAG antibody, and qPCR was carried out with the primers listed in Supplementary Table S2. *WT* kiwifruit was used as control, and the relative value is set to 1. (E) Y1H analysis, using *LacZ:: AcABI5apro* as the baits, pB42AD::AcABI5a as the prey, with *LacZ::P53pro* and pB42AD::P53 serving as the positive control. (F) EMSA showing the interaction between fusion protein AcABI5a-HIS and the *AcABI5a* promoter. (G) Dual-LUC using *AcABI5apro::LUC* as a reporter and *35S::AcABI5a* as the effector, *N. benthamiana* leaves were cotransformed with the reporter, with the empty effector vector used for the negative control. (H) ChIP assay of *OE-AcABI5a::FLAG* transgenic kiwifruit leaves to show the binding of AcABI5a to the G-box (CACGTC) of *AcABI5a* promoter DNA. The chromatin was immunoprecipitated with FLAG antibody, and qPCR was carried out with the primers listed in Supplementary Table S2. *WT* kiwifruit was used as control, and the relative value is set to 1. These experiments were repeated three times each. The error bars indicate the SD values from three biological repetitions. Significant differences were detected by *t*-test (^**^P < 0.01, ^***^P < 0.001).

A yeast one-hybrid (Y1H) assay was used to test the interaction between AcABI5a and the promoter of *AcMYBS1*. Yeast cells cotransformed with the *AcMYBS1* promoter and *AcABI5a* gene exhibited normal growth and displayed blue coloration on SD/−Trp/Ura/BU salt+X-gal medium (Fig. 4A), indicating binding of AcABI5a to the *AcMYBS1* promoter. As ABI TFs are recognized for their affinity to G-box elements [23], electrophoretic mobility shift assays (EMSA) were performed, and the assays confirmed binding of AcABI5a to the *AcMYBS1* promoter. Clear bands were evident when AcABI5a protein was added to *AcMYBS1* probes containing the G-box element, and this weakened upon addition of an unlabeled competitor and were absent when mutant probes replaced 6-carboxyfluorescein (FAM) labelled probes (Fig. 4B). Dual-luciferase reporter assays in *N. benthamiana* leaves further demonstrated that coexpression of AcABI5a and *AcMYBS1* promoter-driving luciferase (LUC) led to a 70% reduction in LUC/renilla (REN) ratio compared with the EV control (Fig. 4C). We performed chromatin immunoprecipitation coupled with quantitative PCR (ChIP-qPCR) on DNA extracted from *OE-AcABI5a::FLAG* transgenic and *WT* leaves of kiwifruit. Indeed, the AcMYBS1 fragment containing the G-box was enriched by immunoprecipitation (Fig. 4D).

As other ABIs have been found to bind their own promoter and self-activate expression [23, 38], we investigated the potential of AcABI5 to do the same using Y1H assays. These showed that yeast cells cotransformed with the *AcABI5a* promoter and *AcABI5a* coding sequence (CDS) exhibited normal growth and blue coloration, unlike those transformed with the EV (Fig. 4E). EMSA assays using FAM probes of the *AcABI5a* promoter fragment containing the G-box motif confirmed direct binding of AcABI5a to its own promoter, with binding decreasing in the presence of a competitor probe and being absent with mutant probes (Fig. 4F). Dual-luciferase reporter assays revealed ~3.5-fold increase in LUC/REN ratio when the *AcABI5a* promoter and *AcABI5a* CDS were transiently coexpressed in *N. benthamiana* leaves compared with the EV control (Fig. 4G). The ChIP-qPCR assay further demonstrated that AcABI5a did bind to the G-box of the AcABI5a promoter *in vivo* (Fig. 4H). These parallel approaches demonstrate that AcABI5a directly binds to its own promoter and activates transcription.

### AcABI5a interacts with AcMYBS1 *in vivo* and *in vitro*

**Figure 5:**
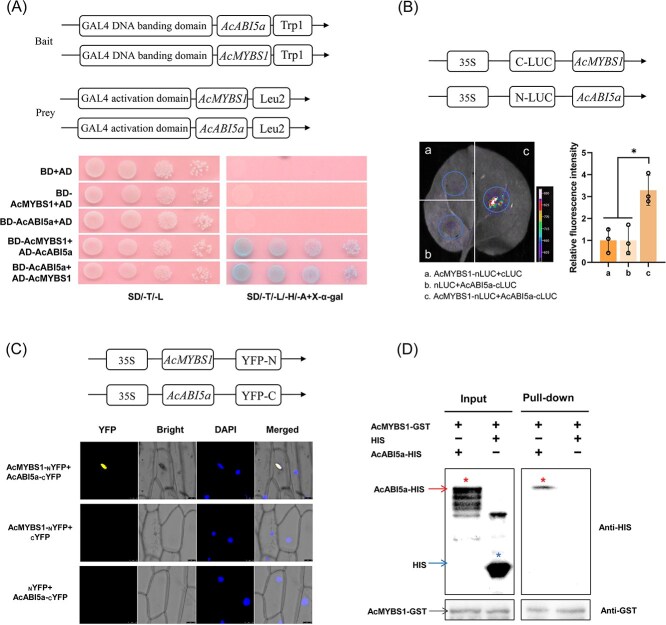
AcMYBS1 interacts with AcABI5a. (A) Y2H analysis, the combinations from the bait BD- AcMYBS1 and prey AD- AcABI5a, bait BD- AcABI5a and prey AD- AcMYBS1, cotransformed into Y2H gold yeast. (B) Analysis of physical interaction of AcMYBS1 and AcABI5a using firefly luciferase complementation assay in *N. benthamiana* leaves. The fluorescence of controls was taken as 1 for normalization, and the representative image was taken. (C) The BiFC assay showed the interaction between AcMYBS1 (_N_YFP) and AcABI5a (_C_YFP). (D) An *in vitro* GST pull-down assay verified the interaction between AcMYBS1 and AcABI5a. AcMYBS1-GST tagged protein was incubated with AcABI5a-HIS or 6 × HIS protein negative control. The top left box shows an Anti-His probed western blot of the input mixture before GST purification. AcABI5a and 6 × HIS protein bands are indicated by arrows. The additional bands present are copurified polyhistidine containing *E. coli* protein contaminants. The pull-down box to the right is the western blot probed with Anti-His antibody after GST purification and shows that only AcABI5a-His bound to the AcMYBS1-GST bait. The boxes below the input and pull-down boxes are duplicate respective westerns probed with Anti-GST antibody- demonstrating the presence of AcMYBS1-GST tagged bait protein in each of the tested mixes. These experiments were repeated three times each. The error bars indicate the SD values from three biological repetitions. Significant differences were detected by *t*-test (^*^P < 0.05).

To determine if AcABI5a and AcMYBS1 protein directly interact, we conducted yeast two-hybrid (Y2H) and firefly luciferase complementation (NC-LUC) assays. Yeast cells expressing AcMYBS1 and AcABI5a could grow and were blue-colored on SD/−Trp/−Leu/-His/−Ade + X-α-gal medium (Fig. 5A). Subsequently, NC-LUC assays tested these findings, with luminescence detected in *N. benthamiana* leaf cells expressing both N-LUC-AcMYBS1 and C-LUC-AcABI5a (Fig. 5B). To ascertain the cellular localization of their interaction, a bimolecular fluorescence complementation (BiFC) assay in onion epidermal cells was conducted, revealing fluorescent signals located exclusively within nuclei containing both AcMYBS1 and AcABI5a (Fig. 5C). Finally, we purified His- and GST-tagged AcMYBS1 and AcABI5a (heterologously expressed in *Escherichia coli*) for use in pull-down assays to verify the interaction between AcMYBS1 and AcABI5a *in vitro*. The results demonstrated that only AcABI5a-HIS protein, and not HIS protein alone, was pulled down by AcMYBS1-GST protein, corroborating the interaction between AcMYBS1 and AcABI5a, both *in vivo* and *in vitro* (Fig. 5D). These findings establish that AcABI5a and AcMYBS1 can physically interact.

### AcABI5a mediates the ABA signal to inhibit AsA synthesis

**Figure 6:**
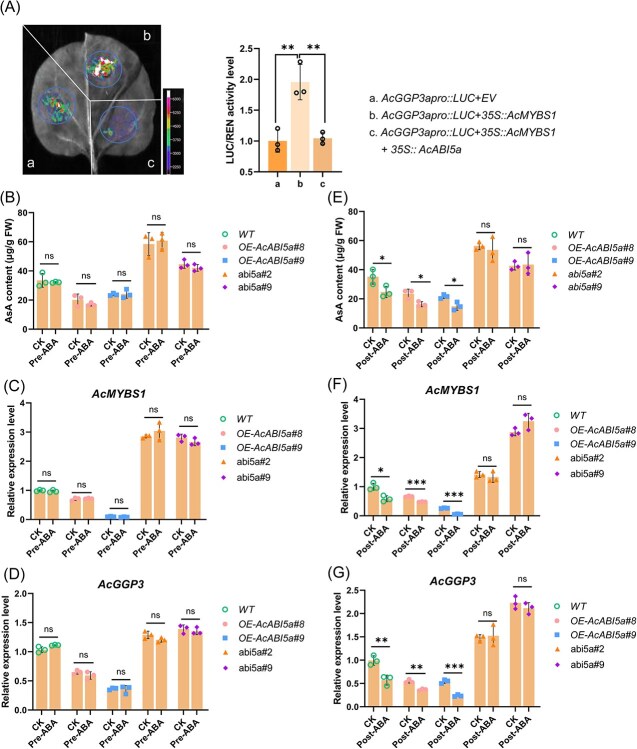
*AcABI5a* mediated an ABA signal to inhibit AsA synthesis in kiwifruit leaves. (A) Dual-LUC using *AcGGP3apro::LUC* as a reporter, *35S::AcMYBS1* and *35S::AcABI5a* as the effectors, *N. benthamiana* leaves were cotransformed with the reporter and the empty effector vector was used as the negative control. LUC/REN ratio of the control was taken as 1 for normalization, and the representative image was taken, and this experiment was repeated three times. (B, C, D) The AsA content (B), expression level of *AcMYBS1* (C), and *AcGGP3* (D) in leaves from plants of *AcABI5a* overexpression and gene editing lines at the start of the experiment before ABA treatment (Pre-ABA). So, for B/C/D ‘Pre-ABA’ is effectively also an untreated control, and ‘CK’ is a control that was never treated with ABA. The AsA content (E), expression level of *AcMYBS1* (F) and *AcGGP3* (G) of *AcABI5a* overexpression and gene editing lines after treatment with 10 μM ABA (Post-ABA). ‘CK’ refers to untreated control. All error bars denote standard deviation (±SD), *n* = 3. Significant differences were detected by *t*-test (^*^P < 0.05; ^**^P < 0.01; ^***^P < 0.001); ns: no significance).

Previous studies have established that AcMYBS1 activates *AcGGP3* transcription and promotes kiwifruit AsA synthesis [6]. To elucidate how AcABI5a interacts with AcMYBS1 to regulate *AcGGP3* transcriptional activity, we performed a Dual-LUC assay in *N. benthamiana*. As before, AcMYBS1 activates the transcriptional activity of the *AcGGP3* promoter. While *35S-*driven AcMYBS1 alone activated *AcGGP3* promoter transcription, coexpression with *35S-*driven *AcABI5a* attenuated this activation, suggesting that an *in vivo* interaction between AcABI5a and AcMYBS1 inhibits the ability of AcMYBS1 to activate *AcGGP3* transcription (Fig. 6A).

ABIs are characterized as a key element in the ABA signaling pathway [15, 24]. To investigate the role of AcABI5a in the regulation of kiwifruit AsA synthesis by ABA, tissue culture plantlets of both *AcABI5a-*overexpressing and two gene-edited ‘Donghong’ *Acabi5a* mutant lines were treated with ABA (−ABA: no-ABA treatment; +ABA: treated with 10 μM ABA) or a water control (CK). Before ABA treatment there were no significant differences in leaf AsA content or expression levels of *AcMYBS1* and *AcGGP3* within experimental line pools (Fig. 6B and C and D). The *AcABI5a* overexpression line pools had reduced AsA compared with controls and the edited lines had higher AsA contents. However, post-ABA treatment, both ABA-treated WT and *AcABI5a-*overexpressing line pools exhibited reduced leaf AsA content, *AcMYBS1*, and *AcGGP3* expression, while gene-edited lines showed minimal changes due to loss of ABI5a function (Fig. 6E and F and G). There were some differences in magnitudes between preexperiment measurements and later postexperiment measurements (e.g. Figure 6: panel B vs E; C vs F; and D vs G) and why this is the case is unclear, but it is probably caused by either the natural diurnal variation due to harvest at different times during the day, or else different ages of the explants (as the plantlets were harvested at different dates), or a combination of both.

In the case of overexpressing lines, the further inhibition likely arose from upregulation of endogenous *AcABI5a* suggests that repression driven by *35S* in those lines was not complete. The gene-edited mutants of *AcABI5a* showed that mutating *AcABI5a* can cause just over a doubling of leaf AsA content. These findings show that under normal growth conditions, *AcABI5a* dynamically responds to an increase in ABA to inhibit *AcMYBS1* expression, which in turn inhibits the transcription of *AcGGP3* and results in reduced AsA synthesis.

### Discussion

AsA is known to be a potent nonenzymatic antioxidant and a key coenzyme factor [39–41], and it has garnered interest as a potential anticancer agent [42]. Due to mutations in the last enzyme of the AsA biosynthesis pathway, certain animals, including humans, must acquire AsA through dietary sources to maintain necessary metabolic functions and oxidative defense [43]. Consequently, there is an increasing research focus on the regulation mechanisms of AsA in food, especially in horticultural fruits and vegetables [44–46]. For example, in AsA-rich kiwifruit, PosF21 and MYBS1 proteins bind to the promoter of *GGP3,* enhancing its expression, and thereby the amount of AsA produced. Additionally, MYBS1 interacts with the GBF3 protein to further promote AsA synthesis [6, 10]. AsA acts as an antioxidant regulating a variety of physiological processes in plants [18, 47] but the interplay between AsA and ABA in plant growth and development remains poorly understood. Our study observed an inverse relationship between AsA and ABA synthesis in the fruit of kiwifruit during growth and development, showing an inhibitory effect of ABA on AsA synthesis (Fig. 1A). Previous research has demonstrated ABA’s capacity to suppress *MYBS1* expression, thereby limiting AsA synthesis in kiwifruit, as evidenced in overexpression and gene-edited lines [6]. However, the precise mechanism through which ABA suppressed *MYBS1* expression in kiwifruit warranted further investigation.

The ABI gene family encodes AP2/ERF TFs that serve as a pivotal component in the ABA signaling pathway, and thus are instrumental in various plant developmental processes [15, 22–24]. For example, mutants of *Arabidopsis* ABI genes display enhanced salt tolerance [48, 49]. Overexpression of ABI5 leads to failure of seeds to acquire desiccation tolerance [50, 51]. ABA signaling delays flowering by upregulating flowering locus C (FLC) expression [51]. In addition, components of the signaling and metabolic pathways of growth hormone, cytokinin, gibberellins (GA), jasmonates (JA), and oleuropein steroids have been shown to be involved in ABI regulation or regulated by ABI proteins [52]. The ABA signaling pathway inhibits seed germination by upregulating ABI5 [53]. ABA also reduces seed sensitivity to GA through the interaction of ABI5 and *TaRHT* in GA signaling [54]. Moreover, in *Arabidopsis*, ABI4 and AsA regulate growth and defense-related gene expression via the JA signaling pathway [34, 54], and JA itself promotes AsA biosynthesis [55]. While ABI proteins (and by extension, ABA) are acknowledged as critical regulators of AsA metabolism in *Arabidopsis* growth [34], their precise role in fruit AsA biosynthesis remains an area requiring further investigation. Our study revealed a robust negative correlation between *AcABI5a* gene expression and AsA levels (Fig. 1C), as well as the expression of *AcGGP3* and *AcMYBS1* across different fruit growth and developmental stages. Our overexpression and gene editing results also demonstrate that AcABI5 also has a role in regulation of AsA synthesis in vegetative tissues. AcABI5a works by binding to the *AcMYBS1* promoter to inhibiting its transcriptional activity (Fig. 4) as well as physically interacting with AcMYBS1 protein, which inhibited the ability of AcMYBS1 to activate *AcGGP3* expression (Fig. 5). After the application of ABA there was significant suppression of both *AcMYBS1* and *AcGGP3* expression that resulted in reduced AsA content (Fig. 1 and 6). Conversely, the *abi5a* mutation removed AcABI5a-mediated inhibition of AsA synthesis by ABA, and approximately doubled the AsA content in vegetative tissues (Fig. 6).

In addition to transcriptional repression, ABI5 also binds its own promoter [23] to enhance its expression, thereby further contributing to the repression of MYBS1 expression (Fig. 4). Taking all these results into consideration leads to the conclusion that AcABI5a mediates ABA inhibition of AsA synthesis in kiwifruit over the course of fruit development by inhibiting AcMYBS1 expression.

The interplay between ABA and AsA has been primarily explored under abiotic stress conditions such as salinity and drought [7, 8, 32, 33]. ABA levels are typically low under normal conditions, but stress can trigger an increase in ABA synthesis and response [24]. ABA has been found to stimulate AsA synthesis, reduce ROS accumulation, and increase drought tolerance in tomatoes [7, 32]. Conversely, salt stress initially suppresses AsA biosynthesis, leading to ROS accumulation, while the *abi4* mutant in *Arabidopsis* shows increased AsA accumulation and reduced ROS damage [8]. Additionally, ABA treatment reduces AsA synthesis in *Arabidopsis*, and the promotional effects of ABA on ROS accumulation were inhibited in *vtc2* mutants [9]. An exception is that ABA treatment of potato seedlings increased potato *GGP* expression (*StGGP1* and *2*) [56], suggesting that this effect might be tissue-type dependent, stress dependent, or differ between genera. However, in general, findings in the literature imply that ABA and AsA synthesis are generally antagonistic to each other. ABA promotes ROS accumulation [18, 47] because stress conditions increase ABA synthesis and inhibit AsA production, leading to higher ROS accumulation. We speculate that a feedback mechanism exists in that early in abiotic stress, ABA upregulation and AsA inhibition led to increase ROS, which, upon reaching a threshold, necessitates increased AsA synthesis to detoxify any excess ROS.

Under optimal growth conditions the role of ABA in regulating AsA synthesis in fruit has been unclear. Our results showed a negative correlation between ABA and AsA synthesis in kiwifruit during different periods of fruit growth (Fig. 1B). A very similar pattern of ABA accumulation over development was found in *A. chinensis* var. *deliciosa* ‘Hayward’ fruit [57] (no data about AsA content was collected in that study), which gives rise to the idea that variation in ABA accumulation patterns might explain some of the variation between genotypes for fruit AsA content. Our results suggest that ABA, through AcABI5a, inhibits fruit AsA synthesis in a developmentally controlled manner in kiwifruit. This adds AsA to the large number of traits that ABA regulates during fruit development and ripening, which includes pigmentation, aromatics, softening, and sugar accumulation [58–60]. Therefore, we hypothesize that developmental variation in fruit ABA content and ABA-responsive gene structure (allelic variation) and expression patterns over fruit development could be determinants of the variation in fruit AsA that is observed in kiwifruit germplasm, as well as other important fruit-related traits. This is an area that requires more attention and extension to other types of fruit.

## Materials and methods

### Plant materials and ABA treatment

The fruits of the diploid, red-fleshed kiwifruit, *A. chinensis* ‘Donghong’, were harvested from the greenhouse located at Wuhan Botanical Garden, Chinese Academy of Sciences (latitude N30° 32′, longitude E114° 24′). Explants intended for transgenic and gene editing assays were cultured in a sterile environment. After ‘Donghong’ had set fruit, fruit at 30, 70, 90, 120, and 130 days after fruiting (DAF) were gathered and stored at −80°C for extraction and measurement.

For the ABA treatment assay in ‘Donghong’ fruit, DAF80 fruit were injected with 10 μM ABA and solvent (pure water, CK) used as control. Younger fruit is difficult to infiltrate due to the tissue being firmer and denser, while at 80 days the fruit material is more amenable for this type of experiment. For the ABA treatment assay in explant materials and transgenic plants, both the control and transgenic lines were grown in MS medium, with or without 10 μM ABA. All the samples of ABA treatment were incubated at room temperature (25°C–26°C) under normal humidity levels (40%–60%) for 3 days before being harvested for further experimentation. Growth conditions for ‘Donghong’ plants and *N. benthamiana* entailed 12 h light/12 h dark at 23°C–25°C.

### Measurement of AsA content by high-performance liquid chromatography

The AsA content in ‘Donghong’ kiwifruit and leaves was determined using high-performance liquid chromatography (HPLC), following the method described in a previous study [61]. Fruits and leaves were ground into a fine powder using liquid nitrogen. An appropriate amount of the powdered sample was weighed, and its mass was recorded. The samples were then extracted with 2 ml of 0.1% (w/v) metaphosphoric acid under low-temperature and dark conditions to prevent AsA degradation. The pH of the extract was adjusted to ~5.0–6.0 using NaOH. The solution was subsequently treated with 5 mM DL-dithiothreitol (DTT) for 30 min at room temperature to stabilize AsA. After centrifugation, the supernatant was filtered for analysis. AsA content was quantified using an Accela 1250 HPLC system equipped with a monomeric C18 column (WONDASIL C18, 5 μm, 4.6 × 150 mm; GL Sciences Inc., China). The flow rate was set at 0.5 ml/min, and the injection volume was 10 μl. A calibration curve was generated using standard AsA samples, and the AsA content in the test samples was calculated based on this reference curve.

### Measurement of ABA content

The ABA content of ‘Donghong’ kiwifruit at various developmental stages was measured by Wuhan Metware Biotechnology Co., Ltd. (Wuhan, China; www.metware.cn) using an AB Sciex QTRAP 6500 LC tandem mass spectrometry (LC-MS/MS) platform, following the method described by Miao et al [62]. Fruit tissues were ground into powder using liquid nitrogen, and 50 ± 0.1 mg of the powdered sample was extracted with 1 ml of methanol/ddH2O/formic acid (15:4:1, v/v/v). The mixture was centrifuged at 15 000× g at 4°C for 5 min, and the supernatant was transferred to a new centrifuge tube, concentrated, and redissolved in 100 μl of an 80% methanol/water solution. The solution was then filtered through a 0.22-μm membrane filter. ABA content was determined using Ultra-Performance Liquid Chromatography (UPLC, ExionLC™ AD) coupled with MS/MS (QTRAP® 6500+). The UPLC and MS/MS acquisition conditions were based on the protocols of Niu [63] and Šimura [64], respectively.

### RNA extraction and real-time qualitative PCR analysis

The total RNA of ‘Donghong’ fruit and leaves was extracted by using a Repure Plant RNA Kit (Magen, Guangzhou, China) as described previously [65]. The first-stand cDNA was used as a template for RT-qPCR using the HiScript III All-in-one RT SuperMix Perfect for qPCR and ChamQ Blue Universal SYBR qPCR Master Mix (Vazyme, Nanjing, China). Reactions were performed at 95°C for 30 s, followed by 40 cycles of 10 s at 95°C and 15 s at 60°C, and were analyzed using an Applied Biosystems 7500 Rapid Real-Time PCR System (QuantStudio 6 Flex, Carlsbad, CA, USA) at 95°C for 15 s, 60°C for 1 min, 30 s at 95°C, and 15 s at 60°C for melt curve analysis. The 2^-△△Ct^ method was employed with Achn107181 (kiwifruit actin gene) as the reference gene control. The primers used in this assay are shown in Supplementary Table S2.

### Transcriptome analysis

High-quality total RNA was extracted from each plant sample and was further used to construct the cDNA sequencing library. The cDNA libraries were sequenced on the Illumina platform following the standard protocol. The adapters and low-quality reads were trimmed and filtered from the raw reads. Clean reads were mapped to the ‘Hongyang’ v3 [66] reference genome using the HISAT2 [67]. The gene expression level was quantified with FPKM (Fragments Per Kilobase of exon model per Million mapped fragments) using the Stringtie [68]. DEGs between the experimental group and the blank group were identified using R package DESeq2 with fold change >2 and *P*-value <0.05. Subsequently, GO enrichment analysis of DEGs was performed using the TBtools [69].

### Construction of overexpression and gene-editing vectors

The CDS of *AcABI5a* and *AcMYBS1* (without stop codon) was cloned from cDNA of ‘Donghong’ and inserted into pOE-3 × Flag-DN overexpression vector to obtain *35S::AcABI5a* and *35S::AcMYBS1*, respectively. Editing of *AcABI5a* by CRISPR/Cas9 system was carried out as previously reported [6]. The sgRNA were selected using CRISPR RGEN Tools (http://www.rgenome.net/?tdsourcetag=s_pcqq_aiomsg) and cloned into pPTG-gRNA-Cas9-U6–26 vector [70] to generate *Cas9-AcABI5a*. The primers used for overexpression and the gene-editing vector construction are listed in the Supplementary Table S2.

### Production of transgenic kiwifruit plants

‘Donghong’ was transformed with the *35S::AcABI5a* and *Cas9-AcABI5a* vector separately using *A. tumefaciens* EHA105 as described previously [10]. ‘Donghong’ leaves were chopped, immersed in *A. tumefaciens* solution containing *35S::AcABI5a* or *Cas9-AcABI5a* vector, and shaken for 15 min, washed with sterile water, then incubated for 3 days in darkness in MS medium containing 100 μM acetylsyringone. The transgenic plants were selected in medium containing 75 ~ 100 ~ 125 ~ 150 mg/ml G418 under long-day conditions (16 h light/8 h dark), and the selection medium was replaced every 15 days. The transgenic lines were verified using PCR sequencing and RT-qPCR analysis and the sequences for these are listed in Supplemental Table S2.

### Virus-induced gene silencing and transient expression in kiwifruit

The 400-bp CDS of *AcABI5a* was cloned into the TRV2 vector to obtain TRV-*AcABI5a* [10]. Additionally, TRV-*AcABI5a* and *35S::AcABI5a* vectors were transferred into *A. tumefaciens* EHA105 strain. The *A. tumefaciens* culture was adjusted to OD_600_ = 0.8 and introduced into ‘Donghong’ fruit according to previously described methods [6]. The fruit was incubated at 26°C–28°C. Five days after infection, the fruit were harvested for analysis of both gene expression and AsA content.

### Dual-LUC assay

Approximately 2000 bp of promoter region upstream from start codon ATG of *AcABI5a* and *AcMYBS1* were PCR amplified from ‘Donghong’ DNA and cloned into pGreen II 0800 vector to generate reporter vectors *AcABI5apro::LUC* and *AcMYBS1pro::LUC*, respectively. The *35S::AcABI5a* and *35S::AcMYBS1 pOE-3 × Flag-DN* overexpression vectors described previously were used as effector vectors. The reporter vectors and effector vectors were transferred into *A. tumefaciens* EHA105 cells (containing *pSoup* helper plasmid). The positive transformant was resuspended in the infiltration buffer to an OD_600_ of 0.8, and reporter vectors and effector vectors were mixed in 1:5 (v/v) ratio. Transient transformation and LUC/REN value of Dual-LUC assay in 4-week-old *N. benthamiana* were performed as previously described [6].

### Y1H assay

For Y1H assay, the ~2000-bp promoter of *AcABI5a* and *AcMYBS1* was inserted into pLacZi vector to obtain *Lacz:: AcABI5apro* and *Lacz:: AcMYBS1pro*, respectively. The CDSs of *AcABI5a* were cloned into pB42AD vector to generate pB42AD:: AcABI5a. The pB42AD:: AcABI5a and *Lacz:: AcABI5apro* or *Lacz:: AcMYBS1pro* cotransferred into EGY48 yeast cells that grew in a synthetic and defined medium lacking Ura and Trp (SD/-Ura/−Trp). The positive yeast cells were screened in SD/−T/-U/BU salt+ X-gal medium, then grew in 30°C for 3–5 days, and the specific experimental steps were performed as previously described [10].

### EMSA assay

The full-length CDS (without stop codon) of *AcABI5a* was cloned into pET-32a vector containing 6◊His tag at the both N- and C-terminus to obtain AcABI5a-His protein (65 kDa), and the vector was transferred into *Escherichia coli* BL21 (*DE3*) competent cells (TransGene, Beijing, China) to express AcABI5a-His fusion protein, which was purified for use in the EMSA assay. Empty pET-32a vector was also expressed to provide a negative control 6 x His-tagged protein (20.4 kDa). Extraction and purification of the proteins were based on the methods previously described [6]. The AcABI5a purified eluate contained copurified nonspecific *E. coli* poly-Histidine-containing proteins (as seen in Fig. 5D), which normally reflects poor expression/low protein stability, and suggests this protein is difficult to heterologously express in *E. coli* in an unoptimized state. While not desirable, these contaminants were not hypothesized to affect the planned assays so the following assays used semipure AcABI5a. Purified AcABI5a-His fusion protein was incubated with FAM-labeled DNA probes, mutant probes, or competing probes, followed by electrophoresis in polyacrylamide gels, and the fusion proteins were analyzed for binding to the probes using a Multifunctional Laser Imager (FLA9500, Sweden). The specific experimental manipulations of EMSA assay are referred to Hou et al [71].

### ChIP-qPCR

ChIP-qPCR assays were performed as described previously [10]. Briefly, WT and *OE-AcABI5a::FLAG* leaves of ‘Donghong’ were cross-linked in 1% formaldehyde for 20 min, after which the cell nuclei were then isolated and resuspended in high-salt nuclear lysis buffer. Chromatin was sonicated to fragment it and then the protein–DNA complexes were immunoprecipitated with mouse FLAG antibody. After elution and reverse cross-linking, the enriched DNA was purified and assayed by qPCR. The primers used in this assay were listed in Supplemental Table S2.

### Y2H assay

The assay was conducted using a Y2H system (Clontech Laboratories). The CDS of *AcMYBS1* and *AcABI5a* were inserted into pGBKT7 (BD) and pGADT7 (AD) vectors, respectively. Yeast suspensions of different combinations (BD*-AcMYBS1*/AD*-AcABI5a*, BD*-AcMYBS1*/AD, BD*-AcABI5a*/AD*-AcMYBS1*, BD*-AcABI5a*/AD) were cotransformed into Y2H Gold yeast competent cells [4]. The transformants were placed on SD/−Trp/−Leu medium for 3–5 days, and then transferred to SD/−Trp/−Leu/-His/−Ade/X-α-gal medium. The interaction between proteins is determined by whether the yeast develops a blue color or not.

### NC-LUC assay

The firefly luciferase complementation assay was performed as described previously [10]. Briefly, the entire CDS of *AcMYBS1* (without stop codon) was inserted into pCAMBIA1300-nLUC vector, while the whole CDS of *AcABI5a* was cloned into pCAMBIA1300-cLUC. *Agrobacterium tumefaciens* harboring *AcMYBS1* was mixed with those harboring *AcABI5a* in a 1:1 (v/v) ratio (optical density of each culture at 600 nm (OD_600_) was 0.8) and infiltrated into *N. benthamiana* leaves for 3 days. Analysis of the luminescence images and luciferase activity were referred to in the description by Liu et al [6].

### BiFC assay

The entire CDSs without start code of *AcMYBS1* and *AcABI5a* were cloned into pSPYNE-35S and pSPYCE-35S vectors to produce *AcMYBS1*-_N_YFP and *AcABI5a*-_C_YFP, respectively, then introduced into *A. tumefaciens* (GV3101). *Agrobacterium tumefaciens* cultures containing AcMYBS1-_N_YFP and AcABI5a-_C_YFP were mixed as 1:1 (v/v) (OD_600_ of each culture at 600 nm was 0.8) and cotransferred into onion epidermal cells for 36–48 h [4]. The detection of the fluorescence signal was carried out as described previously [4].

### Pull-down assay

The pull-down assay was carried out as previously described [10] to further test the interaction between AcMYBS1 and AcABI5a *in vitro*. The full-length CDS of *AcMYBS1* (without stop codon) was cloned into the pGEX-2 T vector containing a fused GST tag at the N-terminus to produce AcMYBS1-GST-tagged protein (60 kDa). For GST pull-down assay, AcMYBS1-GST and AcABI5a-His or His negative control (expression described earlier) proteins were mixed 1:1 (v/v, total 20 μl) with 1 ml of binding buffer (50 mM Tris–HCl (pH = 7.5), 100 mM NaCl, 0.25% Triton-X100 (v/v), 35 mM β-mercaptoethanol), and the mixture was vertically mixed at 30 rpm for 3–4 h at 4°C using a BE-1200 vertical mixer (Kylin-Bell, Nantong, China). For the AcMYBS1-GST and AcABI5a-His protein pull-down assay, 50 μl Proteinlso GST Resin (Code#DP201, PTransGen, Beijing, China) was washed twice with binding buffer and then incubated with AcMYBS1-GST and AcABI5a-His protein mixture for 3–4 h at 4°C. The resin pellet was collected and washed four times in 1 ml binding buffer (with 5-min centrifugation steps). 6◊SDS protein loading buffer was added (to 1◊final) to the pre- and post-pull-down mixes and then the samples were denatured by boiling for 10 min and then separated on duplicate 10% SDS-PAGE gels and transferred by western blot method to PVDF Transfer Membrane (XH3631831, Thermo Scientific, USA). After transfer one of each duplicate blots was blocked and then probed with either Anti-His (1:10 000 (v/v), TransGene, Code#HT501, Beijing, China) or Anti-GST antibody (1:10 000 (v/v), TransGene, Code#HT601, Beijing, China). The blocking solution consisted of 1% (w/v) nonfat milk powder in Tris-buffered saline solution with 0.1% (v/v) Tween 20, with the respective antibody probe solutions diluted in this. Washing (3 × 10 min) was performed with Tris-buffered saline solution with 0.1% (v/v) Tween 20. For chemiluminescent detection, the blots were probed with HRP-conjugated Goat Anti-Mouse IgG (H + L) (1:1000 (v/v) Proteintech), washed three times as before, and then incubated with Pierce™ ECL Western Blotting Substrate according to manufacturer instructions. The PVDF transfer membranes were then scanned and analyzed using a Multifunctional Imaging System (FluorChem R, Proteinsimple, USA).

### Total protein extraction from kiwifruit leaves

Approximately 1.0 g of fresh transgenic and WT kiwifruit leaves were frozen in liquid nitrogen and then ground into powder using prechilled mortar and pestles. Approximately 250 mg powder was transferred to a 2-ml centrifuge tube, and 1.5 ml of NB1 buffer (50 mM Tris-MES, 500 mM sucrose, 1 mM MgCl2, 10 mM EDTA, 5 mM DTT, 1 mM PMSF, and complete protease inhibitor cocktail) was added, and the samples were fully lysed by mixing thoroughly and then incubating on ice for 30 min. The samples were centrifuged at 14 000◊g for 30 min at 4°C, and the supernatant was retained and measured for total protein concentration with a BCA Protein Quantitative Kit (G2026-200 T, Servicebio, Wuhan, China). The samples were then adjusted to the same concentration and 20 μl of protein extract per sample were tested by western blot incubated with Anti-DYKDDDDK antibody (1:10 000 (v/v), TransGene, Code#HT201, Beijing, China).

### Subcellular localization assay

The whole CDS of AcABI5a (without stop codon) was inserted into pFGC-eYFP vector to produce the *AcABI5a*-YFP fusion vector, then transformed into *A. tumefaciens* (GV3101) and cultured to OD_600_ = 0.8, and then injected into the leaves of 4-week-old *N. benthamiana* plants as described previously [6]. The fluorescence was observed using Confocal Microscopy (Leica TCS-SP8).

### Statistical analysis

Statistical analysis was conducted using a minimum of three biological replications. Significance levels were determined via *t*-tests performed with GraphPad Prism 8.0 software, with significance denoted as follows: ^*^P < 0.05; ^**^P < 0.01; and ^***^P < 0.001. Groupings of significance (*P* < 0.05) were visually represented by distinct letters above the bars in the figures, analyzed using analysis of variance (ANOVA) via SPSS v20. Error bars on the figures depict standard deviations (±SD).

## Supplementary Material

Web_Material_uhaf111

## Data Availability

All the relevant data supporting the findings of this study are available in the paper and supplementary data. The sequence data in this study can be found in the GenBank data libraries under accession numbers were listed in Supplementary Table S1. The sequencing data are available at the NCBI SRA database under accession number PRJNA1126052.
