# Supplementary material for: AcABI5a integrates abscisic acid signaling to developmentally modulate fruit ascorbic acid biosynthesis in kiwifruit
Source: Hortic Res. 2025 Apr 24;12(8):uhaf111. doi: 10.1093/hr/uhaf111 (PMC12258035; doi:10.1093/hr/uhaf111)
Supplement: Web_Material_uhaf111 [file web_material_uhaf111.zip › Liu et al AcABI5a- Supplemental Figures.docx]

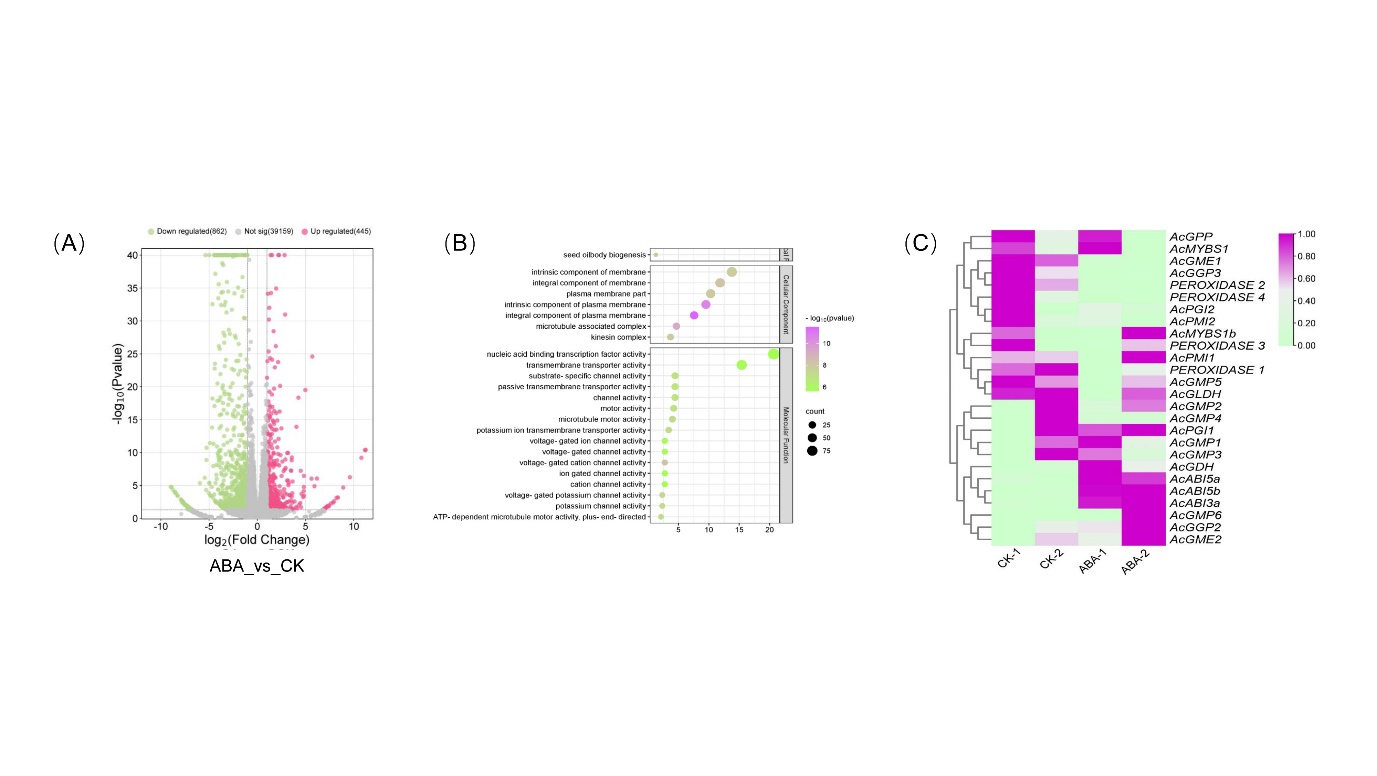


Supplemental Fig. S1. Transcriptome analysis of kiwifruit with ABA or without ABA (CK) treatment. (A) Volcano plot of ABA_vs_CK. (B) GO enrichment analysis of DEGs from transcriptome assay of ‘Donghong’ with ABA treatment and water control (CK). (C) Expression profiles of ‘Donghong’ fruits under ABA (ABA) or without ABA (CK) treatment.


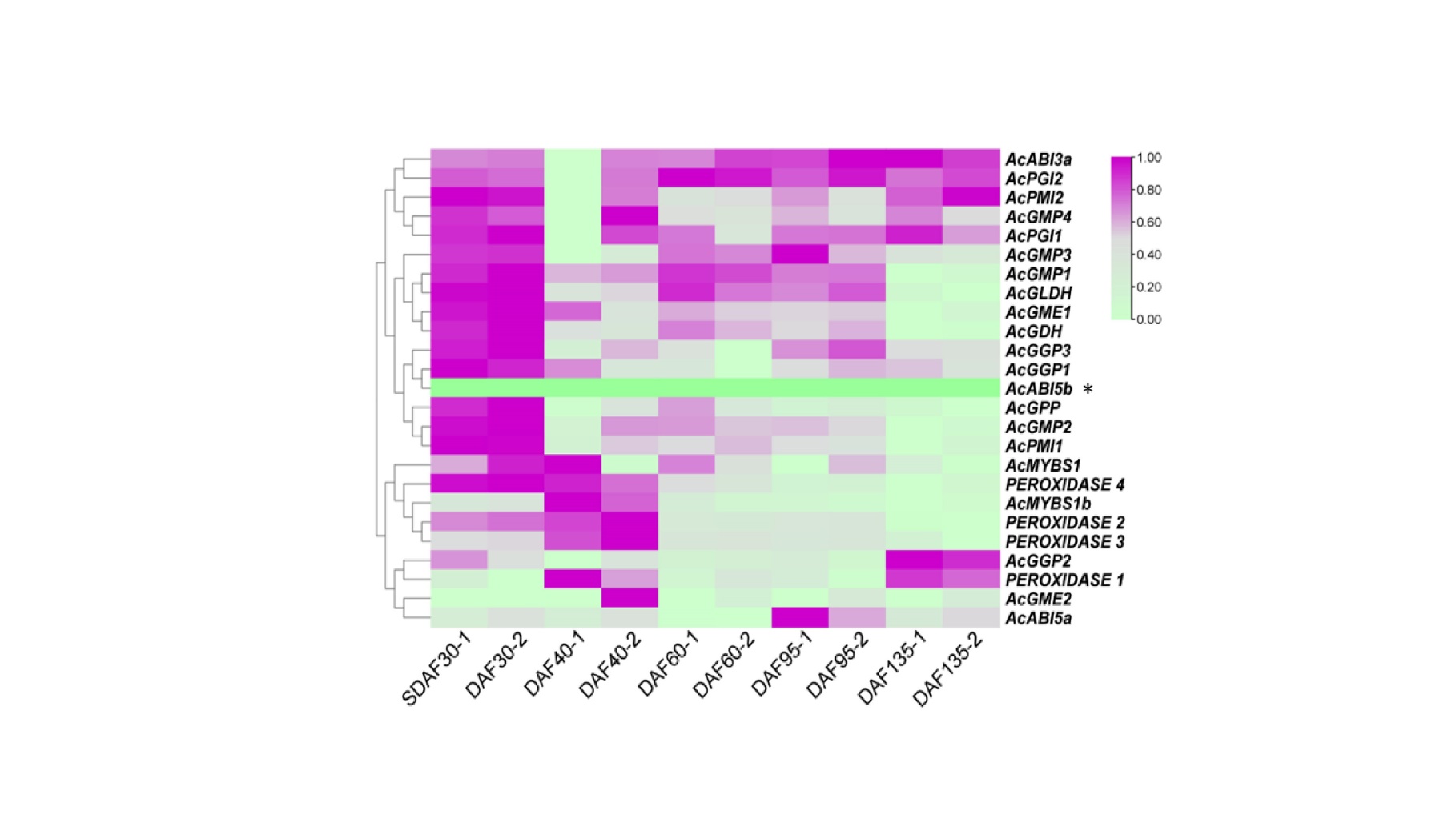


Supplemental Fig. S2. Expression profiles of genes at different development stages in ‘Donghong’ fruits (from DAF30 to DAF135).


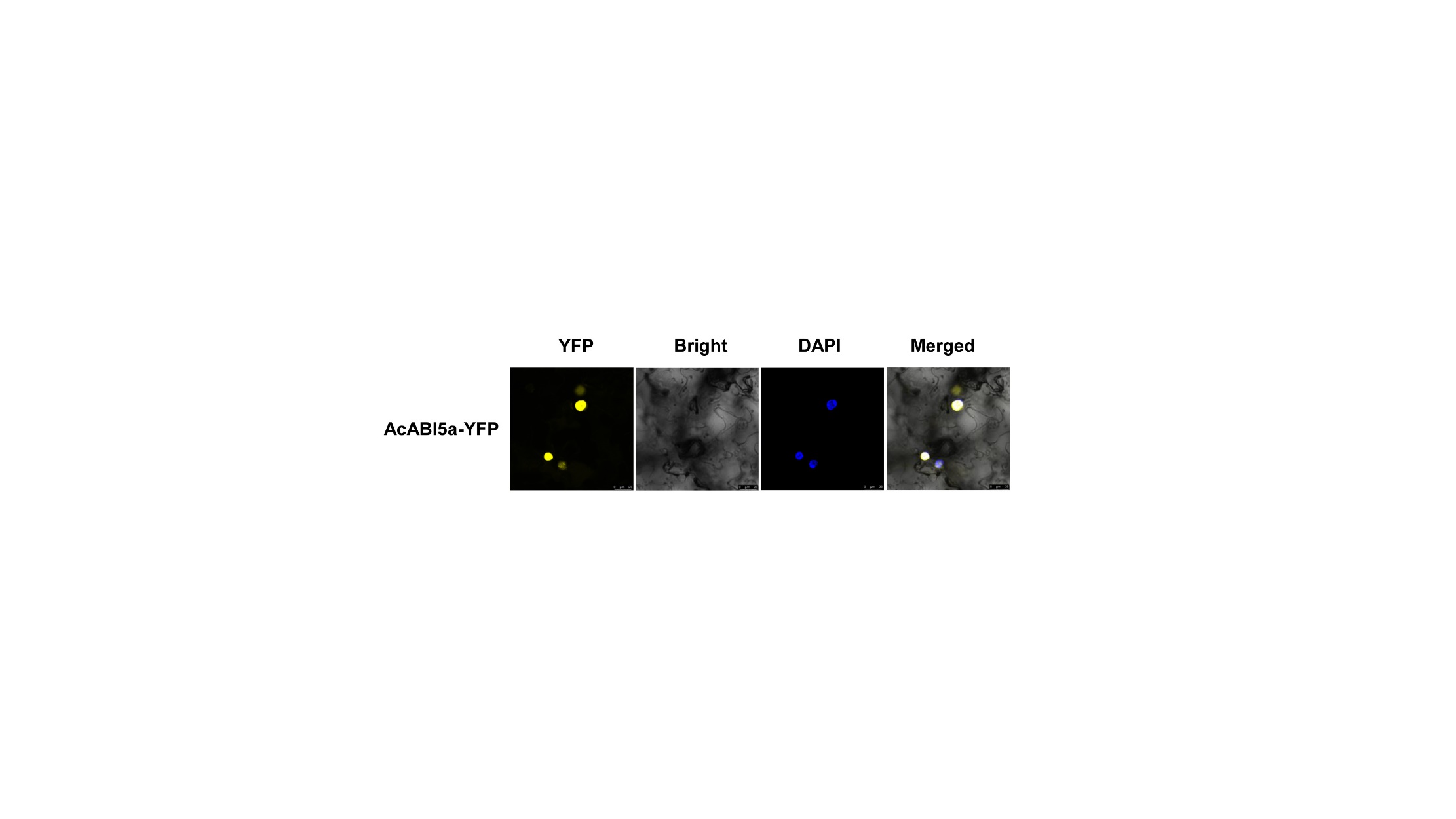


Supplemental Fig. S3. Subcellular localization of AcABI5a in *N. benthamiana* leaves.


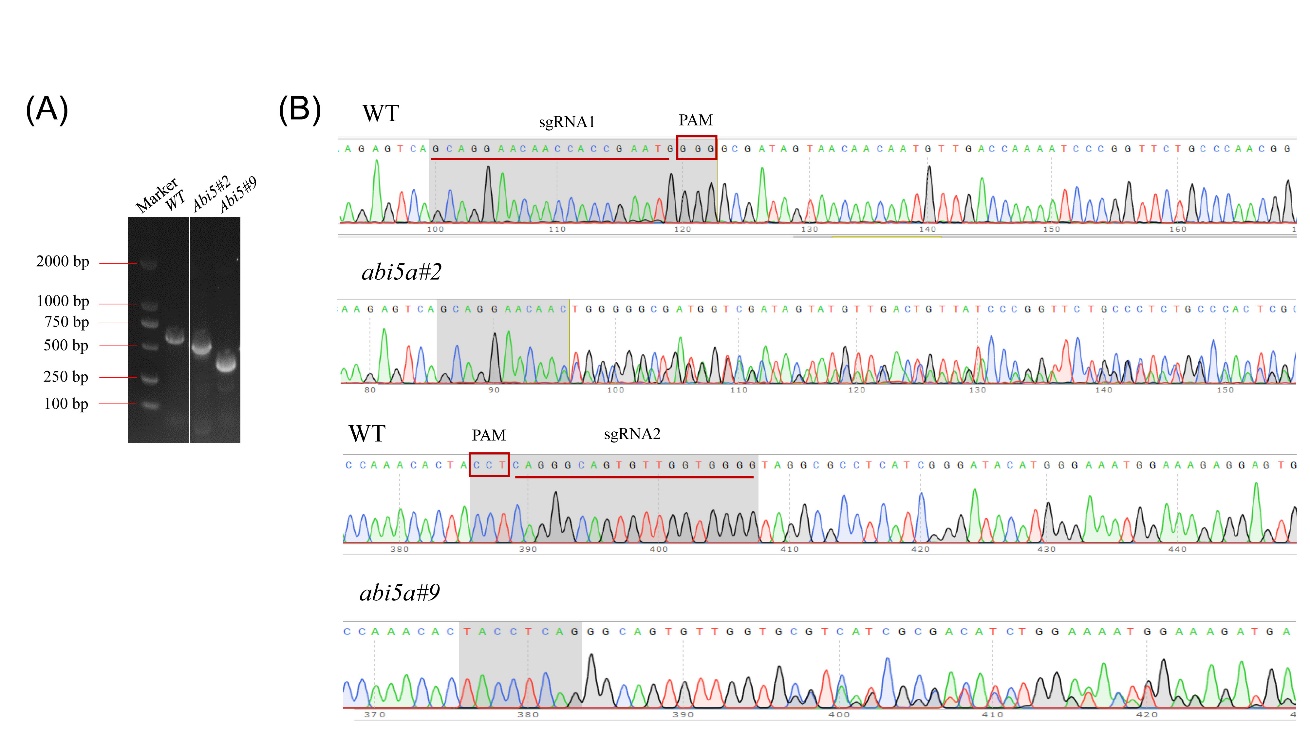


Supplemental Fig. S4. Characterization of *AcABI5a* gene-edited lines. Example of amplification using gene-specific primers, which identified the deletion in AcABI5a in lines *Abi5#2* and *Abi5*#9, the wild-type (*wt*) as a control. (B) Sequences and chromatograms near sgRNA1 and sgRNA2 of the *AcABI5a* gene editing lines.


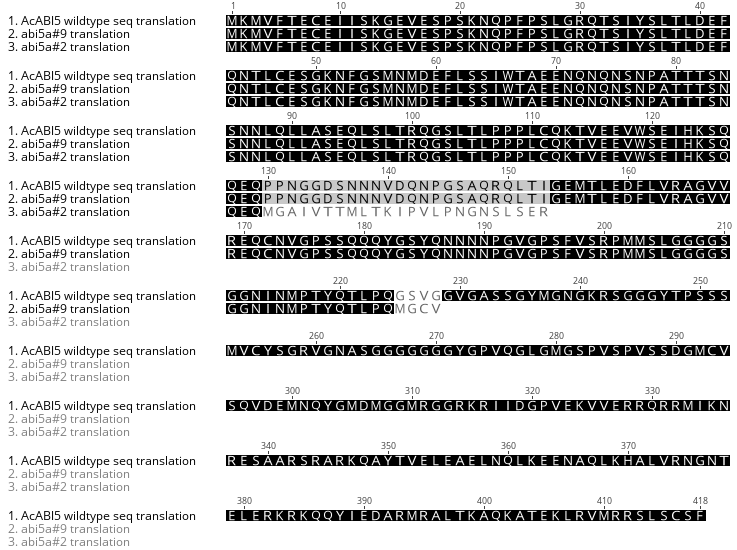


Supplemental Fig. S5. Alignment of coding sequences for the predicted peptide open reading frames for wild type AcABI5 and the two gene edited lines. Image created using Geneious: Geneious version 2022.0 created by Biomatters (https://www.geneious.com).
