# Supplementary figures and images for: AcABI5a integrates abscisic acid signaling to developmentally modulate fruit ascorbic acid biosynthesis in kiwifruit
Source: Hortic Res. 2025 Apr 24;12(8):uhaf111. doi: 10.1093/hr/uhaf111 (PMC12258035; doi:10.1093/hr/uhaf111)

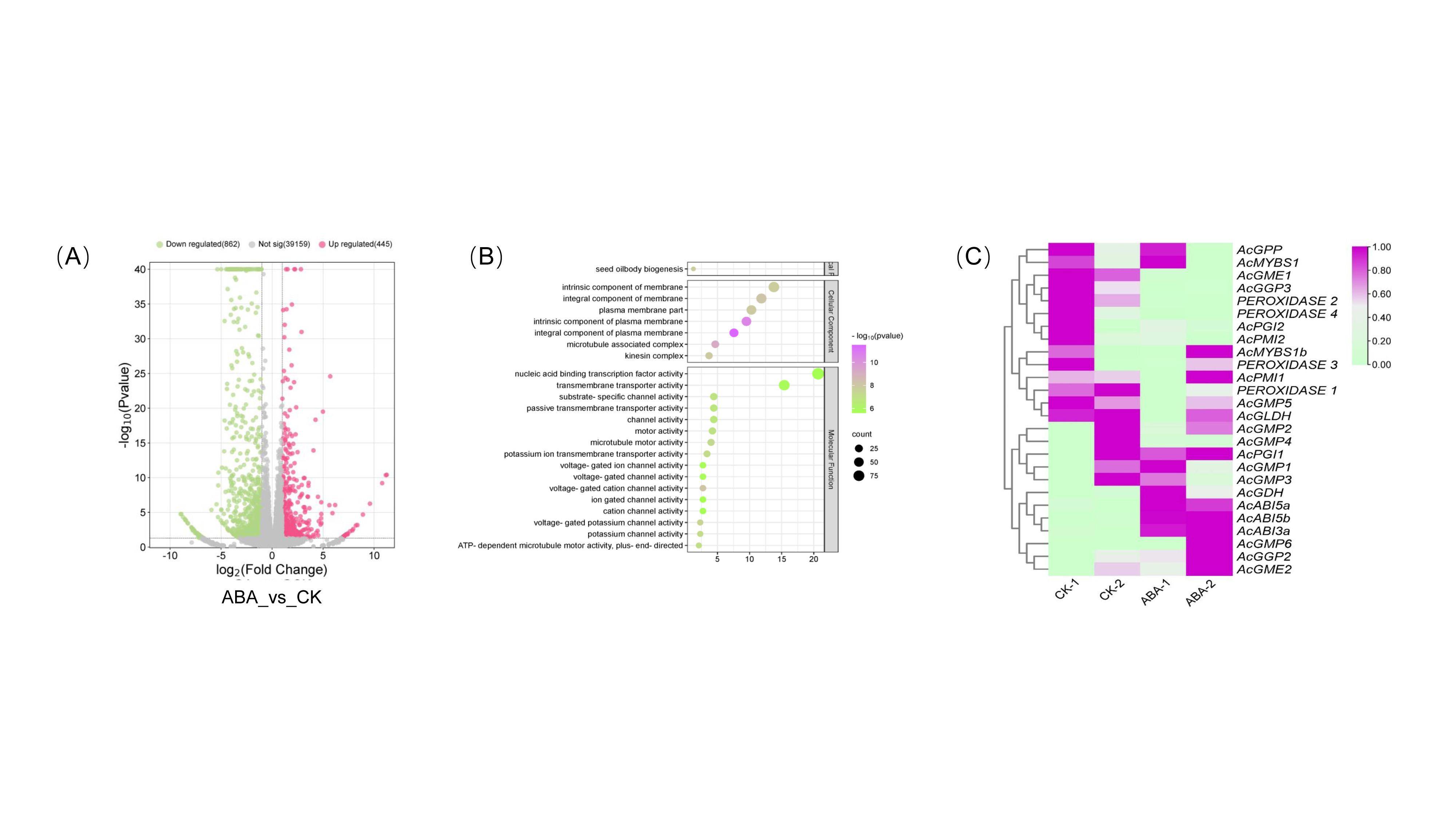

Supplement: Web_Material_uhaf111 [file web_material_uhaf111.zip › Supplemental Figure S1.jpg]

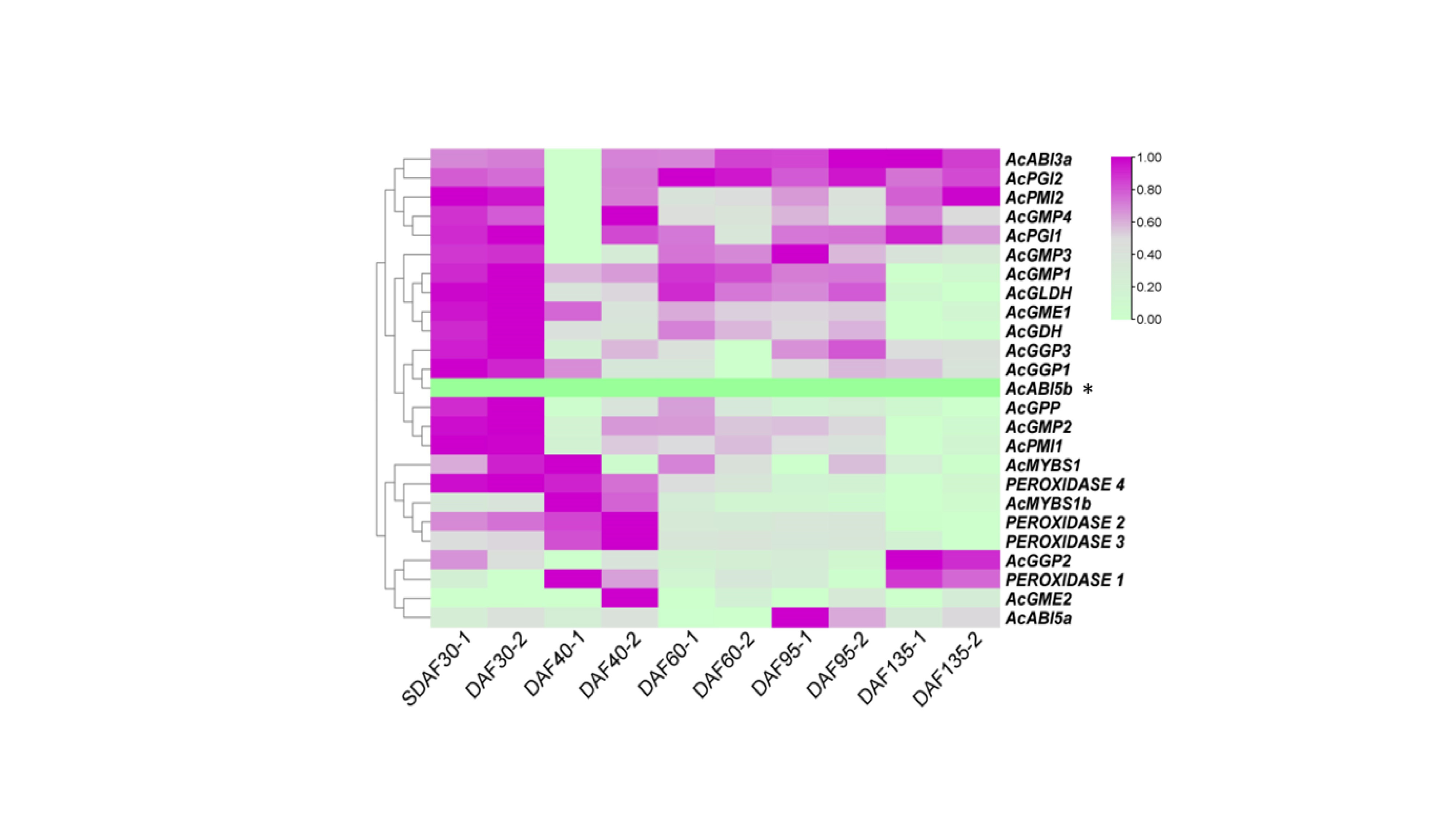

Supplement: Web_Material_uhaf111 [file web_material_uhaf111.zip › Supplemental Figure S2.jpg]

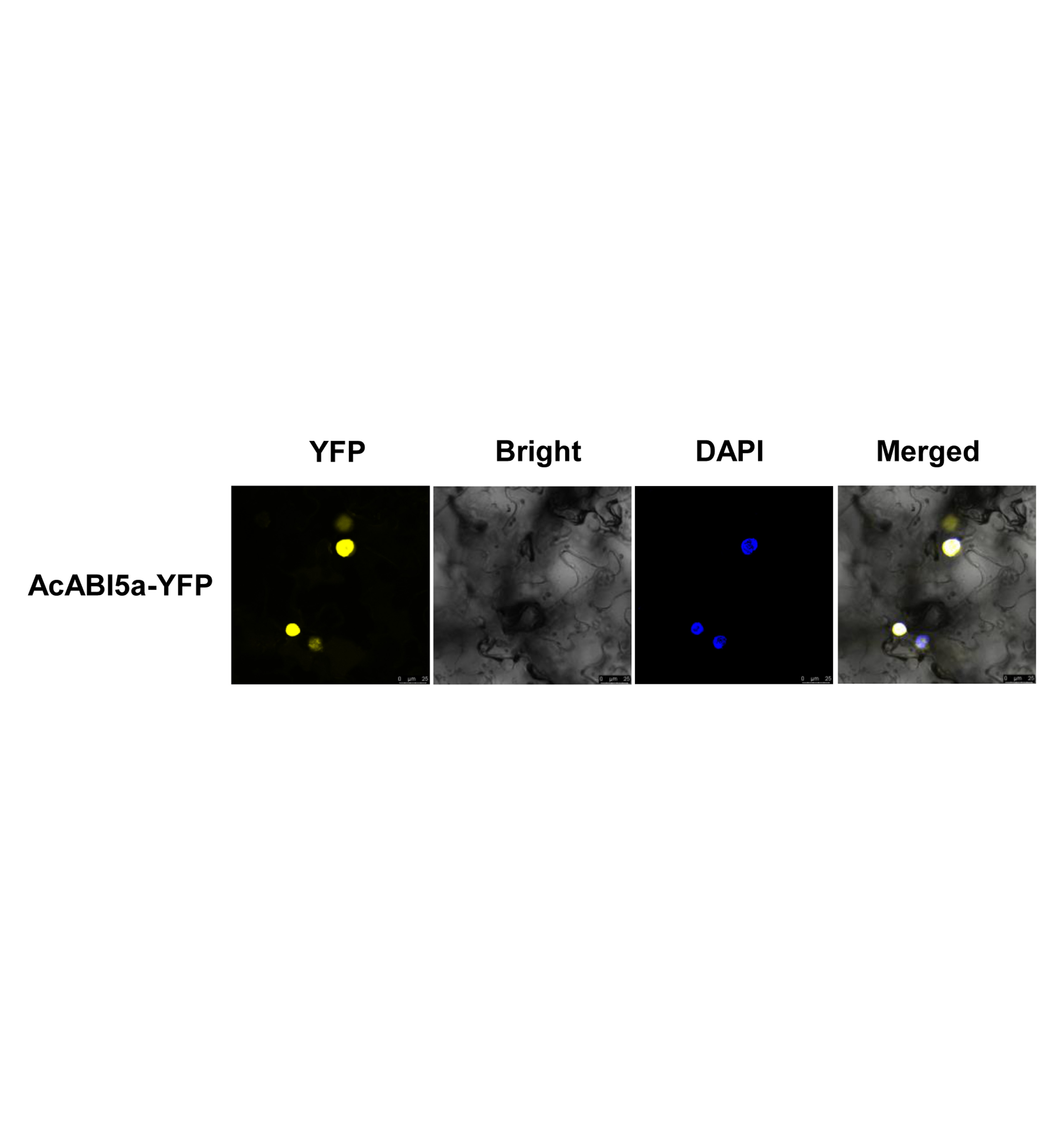

Supplement: Web_Material_uhaf111 [file web_material_uhaf111.zip › Supplemental Figure S3.jpg]

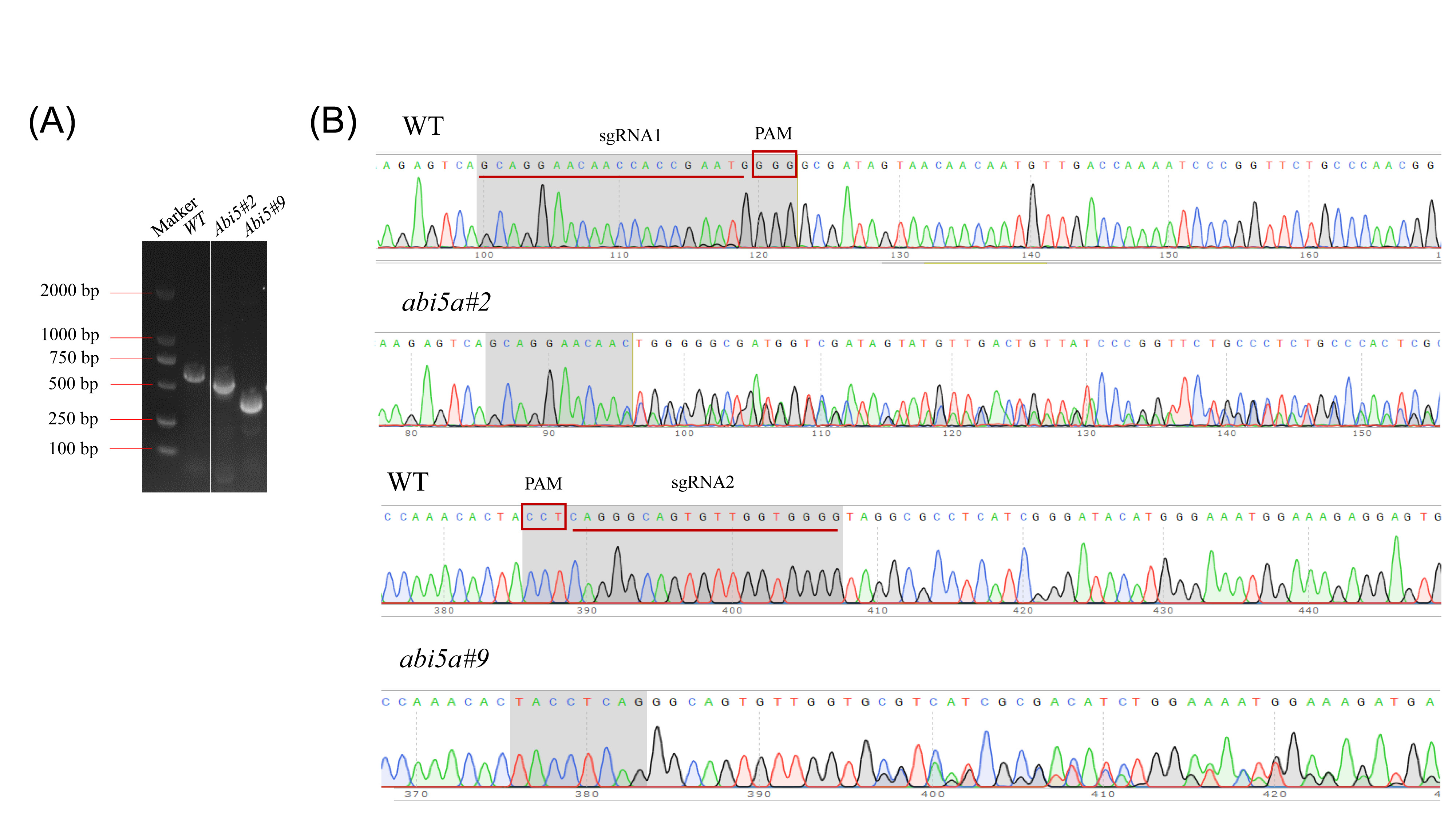

Supplement: Web_Material_uhaf111 [file web_material_uhaf111.zip › Supplemental Figure S4.jpg]

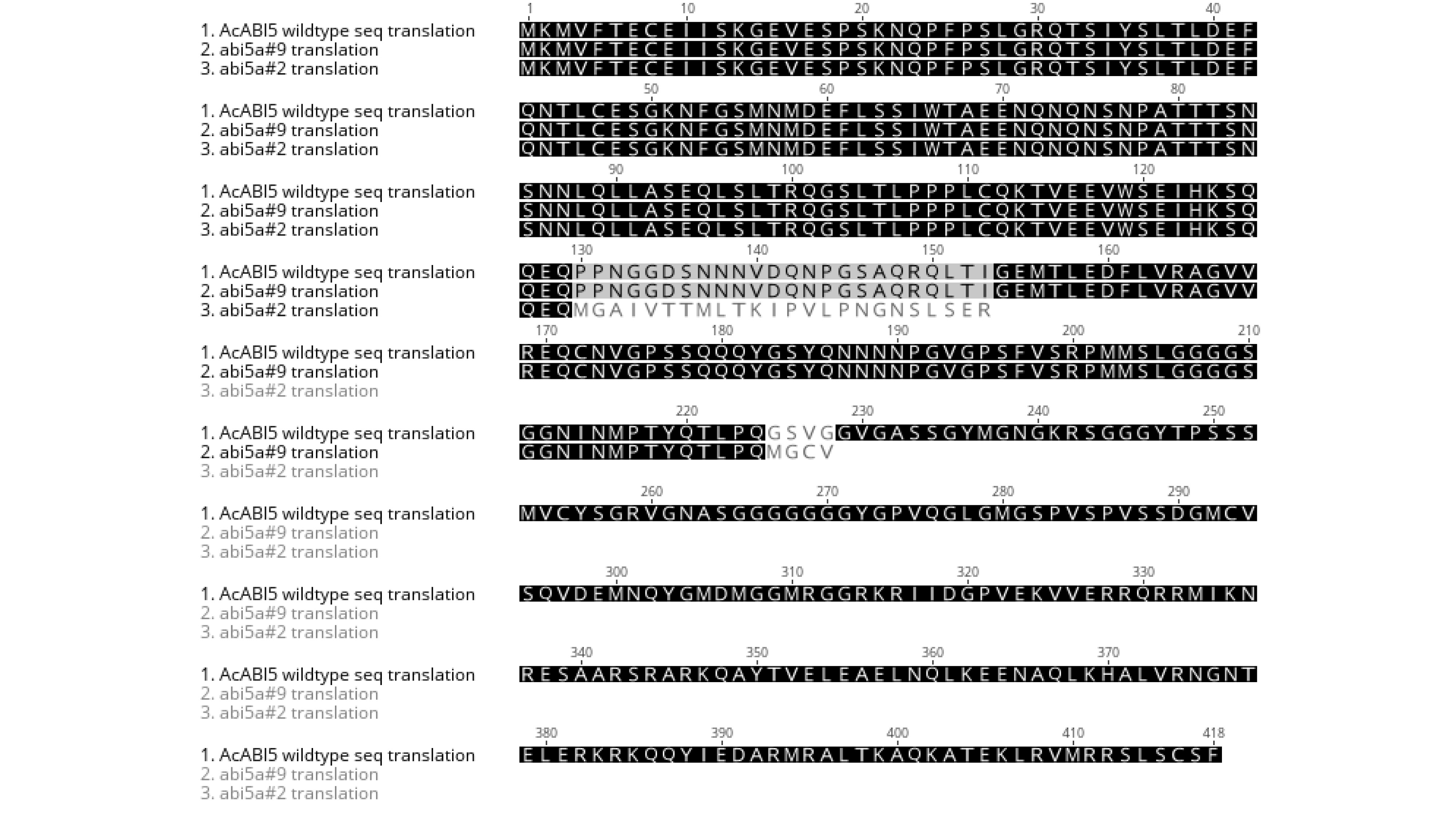

Supplement: Web_Material_uhaf111 [file web_material_uhaf111.zip › Supplemental Figure S5.jpg]
